# Endothelial dysfunction in congenital adrenal hyperplasia due to 21-hydroxylase deficiency: current knowledge and novel biomarkers

**DOI:** 10.3389/fendo.2025.1581681

**Published:** 2025-06-03

**Authors:** Joanna Hubska, Zuzanna Roszkowska, Małgorzata Bobrowicz, Sebastian Iwaniuk, Beata Rak-Makowska, Urszula Ambroziak

**Affiliations:** ^1^ Department of Internal Medicine and Endocrinology, Medical University of Warsaw, Warsaw, Poland; ^2^ Doctoral School of the Medical University of Warsaw, Warsaw, Poland; ^3^ Student Scientific Club “Endocrinus” Affiliated to the Department of Internal Medicine and Endocrinology, Medical University of Warsaw, Warsaw, Poland; ^4^ Laboratory of Experimental Medicine, Medical University of Warsaw, Warsaw, Poland

**Keywords:** congenital adrenal hyperplasia (CAH), 21-hydroxylase deficiency, endothelial dysfunction, endothelial biomarkers, cardiovascular disease, cardiovascular risk

## Abstract

Congenital adrenal hyperplasia (CAH) due to 21-hydroxylase deficiency (21OHD) is a complex endocrine disorder characterized by impaired cortisol synthesis and androgen excess. Beyond its hormonal and metabolic implications, CAH has been increasingly associated with an elevated risk of cardiovascular complications, including endothelial dysfunction, a critical precursor to atherosclerosis and a risk factor for cardiovascular and metabolic diseases. This review explores the current knowledge on endothelial function in patients with CAH, focusing on the interplay between chronic hormonal imbalance, prolonged glucocorticoid treatment, and associated metabolic disorders. We also discuss *in vivo* methods for assessing endothelial function alongside the potential utility of novel biomarkers, which may facilitate earlier identification of vascular dysfunction and stratification of cardiovascular risk. By summarizing emerging concepts in this field, we aim to highlight areas for future research and opportunities for improving long-term cardiovascular outcomes in individuals with 21OHD.

## 1 Introduction

Congenital adrenal hyperplasia (CAH), due to 21-hydroxylase deficiency (21-OHD), is an autosomal recessive condition that is caused by mutations in the gene CYP21A2. It is characterized by impaired cortisol secretion and androgen excess. 21-OHD is the most common cause of CAH, accounting for 95% of cases (1). Based on the residual enzyme activity, CAH shows a spectrum of phenotypes, varying from a severe classic CAH (CCAH), usually diagnosed in newborns, to a non-classic CAH (NCCAH), which is a mild variant often diagnosed late, if ever. CCAH is classified into two forms based on aldosterone deficiency: salt-wasting (SW) and simple virilizing (SV). The primary treatment for CAH, particularly CCAH, involves glucocorticoid and mineralocorticoid replacement to prevent adrenal crises and manage excess androgen production. Achieving a balance between these treatments is essential to avoid both under- and over-treatment, as both extremes can have detrimental effects on long-term metabolic and cardiovascular health. However, even in an era of continuously advancing knowledge about CAH, improved patient care, and the availability of effective treatments, such as those mimicking the circadian rhythm of cortisol, the presence of CAH remains associated with numerous metabolic complications and increased cardiovascular morbidity (2).

Mounting evidence has shown that the dysfunction of endothelial cells in the vasculature is profoundly implicated in the pathogenesis of cardiovascular and metabolic diseases (3). Furthermore, there is a bidirectional relationship between endothelial dysfunction and these disorders. Components of metabolic syndrome, such as abdominal obesity, hypertension, and impaired glycemic control, can contribute to endothelial dysfunction (4). Conversely, structural and functional changes in the endothelium promote the progression of metabolic diseases and atherosclerosis (5). Given the hormonal imbalances and systemic effects of CAH, understanding its potential impact on endothelial function is crucial, as individuals with CAH are at higher risk for these conditions (6, 7).

Despite the growing body of evidence linking CAH to endothelial dysfunction, significant research gaps persist. To date, no studies have systematically assessed the relationship between endothelial dysfunction and glucocorticoid dose, type, or treatment duration. Importantly, the differential impact of various chronic glucocorticoid replacement regimens on endothelial function has not been evaluated in controlled studies (8). Moreover, the progression of endothelial dysfunction over time in individuals with CAH remains insufficiently understood. To date, no randomized controlled trials have been conducted in this area, and no meta-analyses are available to synthesize the existing evidence.

In this review, we provide an overview of current knowledge on endothelial function in individuals with CAH, with a particular focus on the factors that contribute to endothelial damage, methods of endothelial assessment, and novel biomarkers that could help to detect patients at higher risk. We also discuss gaps in knowledge and areas for future research.

## 2 Endothelial dysfunction and its role in cardiovascular disease

The endothelium, a single-cell layer lining the inner surface of blood vessels, plays a vital role in maintaining vascular homeostasis. The endothelium releases various autocrine, paracrine, and endocrine substances, such as nitric oxide (NO), C-type natriuretic peptide, prostacyclin, and endothelium-derived hyperpolarizing factor (9). These factors collectively inhibit smooth muscle cell proliferation and migration, prevent platelet adhesion and aggregation, and regulate processes that influence thrombogenesis (10).

Endothelial dysfunction involves a shift in endothelial cell behavior, leading to various maladaptive changes in their functional phenotype. This results in disturbances in the regulation of hemostasis, thrombosis, vascular tone, redox balance, and inflammatory processes (11). The underlying pathophysiology is multifaceted, involving several mechanisms.

**Figure 1:**
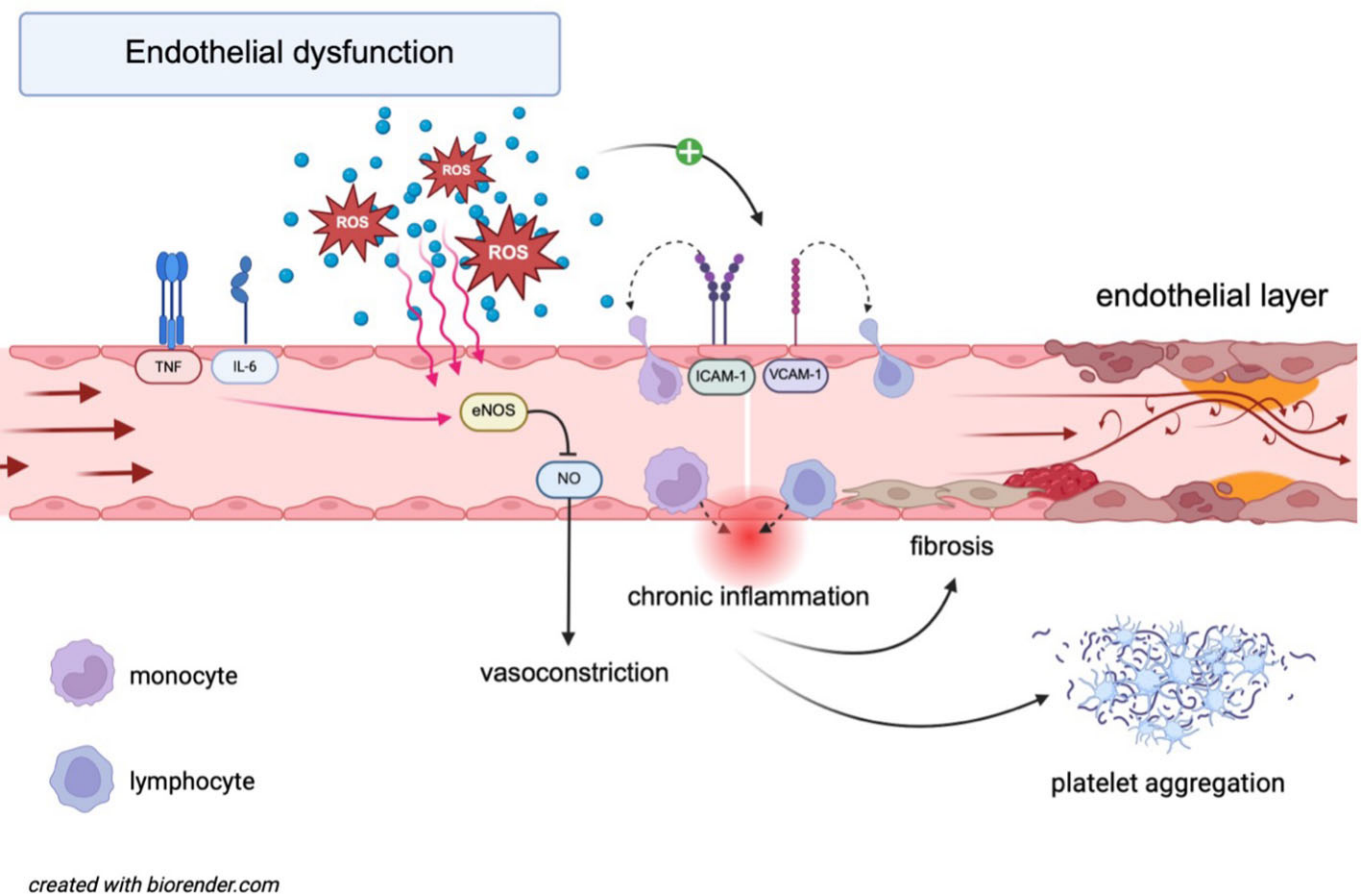
Molecular mechanisms underlying endothelial dysfunction. IL-6 and TNF-α induce eNOS dysfunction, leading to reduced NO bioavailability and vasoconstriction. ROS contribute to oxidative stress, further impairing eNOS activity and promoting chronic inflammation. ROS also upregulate adhesion molecules (ICAM-1, VCAM-1), facilitating monocyte recruitment. This cascade promotes the adhesion and migration of lymphocytes across the endothelial barrier, further activating an inflammatory state. Platelet aggregation is simultaneously enhanced, increasing the risk of thrombosis. Collectively, these processes drive endothelial dysfunction and contribute to the progression of cardiovascular disease. eNOS, endothelial nitric oxide synthase; ICAM-1, intercellular adhesion molecule 1; IL-6, interleukin-6; NO, nitric oxide; ROS, reactive oxygen species; TNF, tumor necrosis factor; VCAM-1, vascular cell adhesion molecule 1.

A key factor in the development of endothelial dysfunction is oxidative stress, which arises from multiple enzymatic sources such as xanthine oxidase, NADPH oxidases, uncoupled endothelial nitric oxide synthase (eNOS), and malfunctioning mitochondria. It occurs when the balance between pro-oxidants and antioxidants is disrupted (12). Elevated reactive oxygen species (ROS) levels can oxidize cellular macromolecules and reduce NO production by promoting the formation of peroxynitrite (12), a toxic compound that degrades the eNOS cofactor tetrahydrobiopterin (13), leading to the “uncoupling” of eNOS and increased oxidative stress. This oxidative imbalance also contributes to impaired endothelial vasodilation and a proinflammatory environment, as well as the upregulation of adhesion molecules such as intercellular adhesion molecule 1 (ICAM-1) and vascular cell adhesion molecule 1 (VCAM-1), along with chemotactic molecules (12) (**Figure 1**).

Inflammation plays a crucial role in the pathogenesis of cardiovascular disease (14). In response to vascular injury, endothelial cells release a variety of inflammatory molecules, including chemokines, interleukin-8, colony-stimulating factors, monocyte chemoattractant protein-1 (MCP-1), adhesion molecules such as ICAM-1 and E-selectin, and growth factors and other inflammatory mediators (15), leading to the attachment of monocytes and their migration into the vessel wall. Monocyte-derived macrophages ingest oxidized low-density lipoprotein (LDL), forming foam cells and fatty streaks, which lead to the development of plaques affecting the coronary arteries, aorta, and carotid arteries, ultimately resulting in atherosclerosis (16). This cascade promotes the adhesion and migration of leukocytes across the endothelial barrier, further activating an inflammatory state (17). In addition, proinflammatory cytokines such as tumor necrosis factor-alpha (TNF-α) and interferon-gamma (IFN-γ) are released by endothelial cells, activating a vicious circle (18).

Endothelial dysfunction is often widespread throughout the body, as individuals with diagnosed atherosclerosis frequently exhibit endothelial dysfunction in peripheral vascular regions that may not yet show overt signs of the disease. It is also observed in those with a family history of early cardiovascular disease despite the absence of other risk factors (19); in individuals with hypertriglyceridemia (20), dyslipidemia (21), nicotine use (22), and insulin resistance (23, 24); first-degree relatives with type 2 diabetes; and elderly patients regardless of the presence of other comorbidities (25). The advancement of endothelial dysfunction is influenced by the severity and duration of established risk factors and the overall risk profile of individual patients (26).

## 3 The risks for endothelial dysfunction in CAH

The exact mechanisms behind the higher prevalence of cardiometabolic risk elements in patients with CAH remain unclear. Nonetheless, both disease-related and treatment-related elements are being discussed. Importantly, cardiovascular disease is the second most common cause of death in patients with CAH after adrenal crisis (27), and is closely linked to endothelial dysfunction (28).

**Figure 2:**
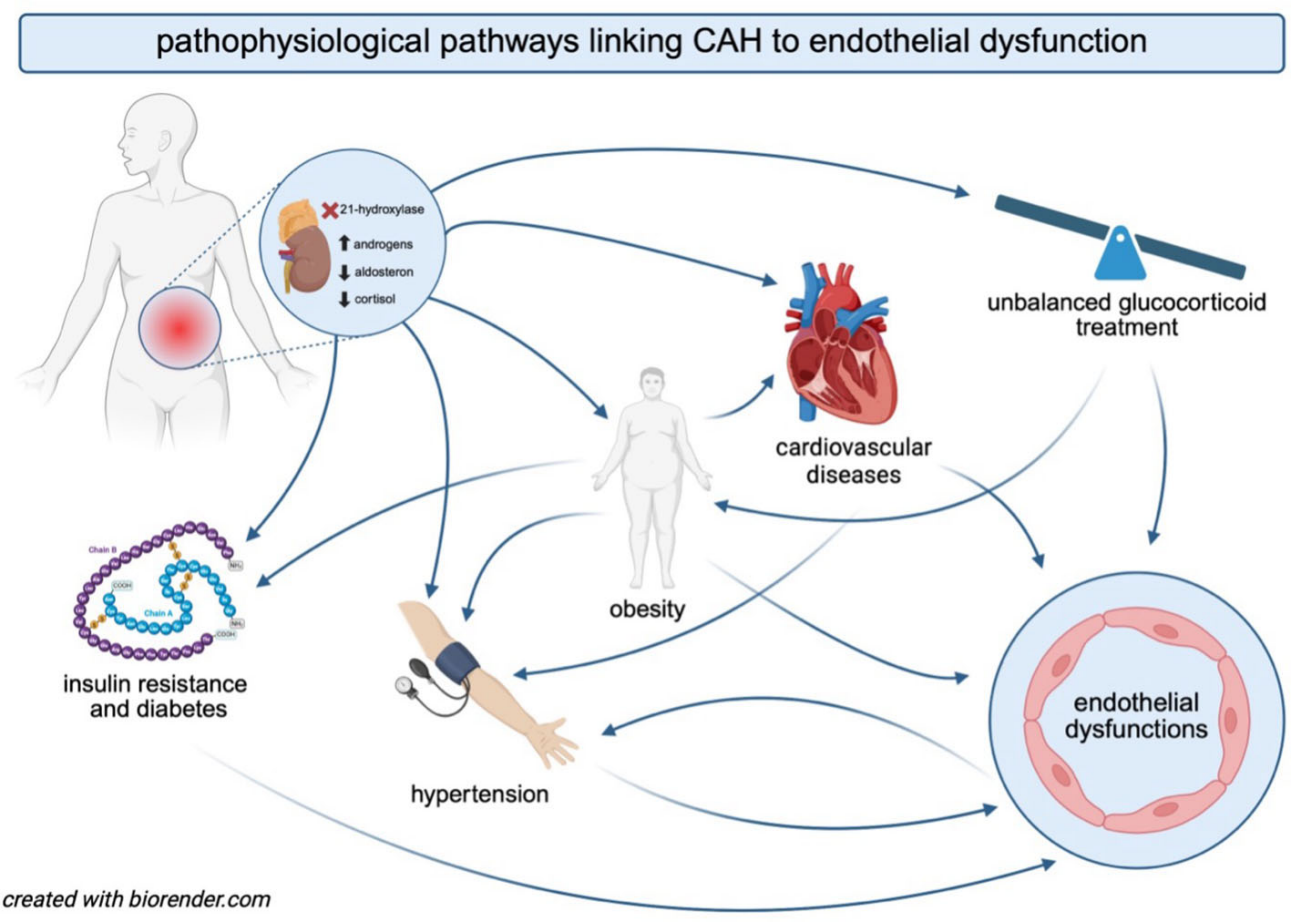
The interplay between CAH, the pathophysiology of comorbidities, and endothelial dysfunction. The figure illustrates the interconnected mechanisms through which CAH contributes to an increased risk of endothelial dysfunction. Hormonal imbalances in CAH promote obesity, insulin resistance, diabetes, and cardiovascular diseases. Glucocorticoid therapy, essential for managing cortisol deficiency in CAH, may further exacerbate endothelial dysfunction and increase cardiovascular risk. Obesity and insulin resistance contribute to the development of hypertension, which, in turn, accelerates endothelial damage and cardiovascular complications. The bidirectional interactions among these factors create a vicious cycle, ultimately predisposing individuals with CAH to an elevated risk of cardiovascular morbidity.

Individuals with CAH are at an increased risk of developing metabolic syndrome, which is characterized by a cluster of metabolic abnormalities, including central obesity, insulin resistance, hypertension, and atherogenic dyslipidemia (29) (**Figure 2**). Obesity is the most common component of metabolic syndrome in both children and adults with CAH and acts as a major independent risk factor for cardiovascular diseases (1). In patients with CAH, the prevalence of obesity ranges from 30% to 40% (30–34). It contributes to endothelial dysfunction through associated complications such as hypertension, dyslipidemia, type 2 diabetes, and obstructive sleep apnea (35, 36). The excess fat accumulation in obesity leads to adipocyte dysfunction, triggering oxidative stress and insulin resistance, while also serving as a source of pro-inflammatory cytokines, all of which contribute to endothelial dysfunction (36, 37). Additionally, patients with CAH are more likely to develop increased visceral adipose tissue, a well-established risk factor for cardiovascular diseases (38).

Notably, endothelial dysfunction is a key component of metabolic syndrome, with involvement in both the initiation and propagation of this condition (39). In patients with CAH, these abnormalities arise from cortisol deficiency, excess androgen secretion, and hypercortisolism due to possible glucocorticoid overtreatment. Additionally, dysfunction of the adrenomedullary system, marked by deficient epinephrine secretion, may contribute to reduced lipolysis of triglyceride stores and disruptions in insulin and adipokine regulation (40–42).

Abnormal androgen levels in CAH should be considered among the risk factors for endothelial dysfunction (**Figure 2**). Both hypoandrogenism in male patients and hyperandrogenism in female patients can contribute to adverse metabolic effects, thereby increasing cardiovascular risk (43–45). Arlt et al. (31) found that the majority of patients with CAH exhibited either elevated or suppressed androgen levels, with only 36% showing normal androstenedione levels. The detrimental effects of androgen excess in CAH were further confirmed in a cohort of women with CCAH SV untreated with glucocorticoids; insulin resistance and unfavorable metabolic markers were notably increased in these patients compared to the control group, and showed a direct correlation to testosterone levels (46). Paizoni et al. (30) also supported the role of androgens in insulin resistance development in women with CCAH, especially in those with poor androgen control. Similarly, there are studies in NCCAH that emphasize the relationship of increased testosterone levels with insulin resistance (47). Moreover, studies in patients with polycystic ovary syndrome (PCOS) have shown a link between hyperandrogenism and impaired endothelial function (48, 49), suggesting that elevated androgen levels may significantly contribute to endothelial dysfunction.

Hypertension is another major risk factor for cardiovascular disease, and in CAH, an increased frequency of hypertension overall is observed across age groups, although more prevalent in children compared to adults (50, 51). Importantly, the relationship between systemic arterial hypertension and endothelial dysfunction is bidirectional, amplifying the severity of both conditions. Endothelial cells influence the development of systemic hypertension through various mediators, while systemic hypertension exacerbates endothelial dysfunction, contributing to a prothrombotic, proinflammatory, and proatherosclerotic state (52). It is well established that excess glucocorticoids can elevate arterial blood pressure mainly through mineralocorticoid mimetic effects, vascular remodeling, and impaired NO signaling (53). However, the role of mineralocorticoid treatment should also be considered; patients with CAH receiving fludrocortisone tend to experience elevated blood pressure more frequently than those who do not (54).

The majority of studies have reported elevated blood pressure values in patients with CAH (32, 55, 56). In youth with CAH, a positive correlation between body mass index (BMI) and blood pressure has been observed (57, 58), highlighting a significant association between hypertension and obesity in this population. Furthermore, even in the absence of clinically overt hypertension (47), individuals with CAH may show a reduced physiological nocturnal dip in blood pressure (30, 59, 60). Gender differences have been examined in a limited number of studies, with most finding a similar prevalence of hypertension between men and women with CAH (32, 61–63). However, two studies suggested that women with CAH may be more affected than men, likely due to excessive androgen exposure (47, 64).

The cardiometabolic status in CAH is strongly affected by the medications used in therapy, as the mainstay of CAH management involves the intake of glucocorticoids and mineralocorticoids (**Figure 2**). The doses should be substitutive; however, patients frequently fail to adhere to the guidelines. Excessive intake of glucocorticoids and mineralocorticoids can raise cardiovascular risk factors, while inadequate glucocorticoid therapy or poor adherence may result in androgen excess, infertility, and the formation of adrenal rest tumors (65, 66). Therefore, preventing long-term metabolic and cardiovascular complications depends on maintaining an optimal balance between overtreatment and undertreatment, however, it remains a significant challenge in both CCAH and NCCAH (67).

Chronic glucocorticoid therapy has been shown to heighten the risk of developing insulin resistance and, subsequently, type 2 diabetes in patients with CAH (2, 31, 32). Adult patients with CAH exhibit elevated fasting plasma glucose levels (31), reduced insulin sensitivity, and a β-cell response that is unable to compensate for insulin resistance (68). Significantly, insulin resistance in CAH seems to be related not only to the cumulative dose of glucocorticoids but also to the type of glucocorticoid used. Patients on long-term dexamethasone show a higher prevalence of insulin resistance compared to those taking prednisolone or hydrocortisone (8).

The impact of glucocorticoids on vascular function, particularly in the context of treatment adherence, remains poorly understood. Non-adherence to treatment in CAH has been correlated with detrimental effects on health, including greater intima–media thickness (69) and a poorer quality of life (70). Finkielstain et al. (32) emphasized the critical importance of consistent treatment adherence in modulating disease outcomes, further highlighting the urgent need for longitudinal investigations in this population.

Interestingly, the effects of systemic glucocorticoid therapy in CAH can also be influenced at the receptor level. Variations in the glucocorticoid receptor gene (NR3C1) may be associated with either negative or positive metabolic and cardiovascular profiles (71). For example, the A3669G polymorphism is linked to unfavorable lipid profiles in pediatric patients with CAH, while the ER22/23EK haplotype reduces glucocorticoid sensitivity, leading to a more favorable metabolic profile. In contrast, the N363S and BclI restriction fragment length polymorphisms increase glucocorticoid sensitivity, raising the risk of type 2 diabetes, obesity, and cardiovascular diseases (72, 73). BclI heterozygotes with CAH show higher body mass index (BMI), waist circumference, and systolic blood pressure compared to those with the wild-type (74), though this polymorphism is less common in patients with CAH than in the general population (71).

## 4 Assessment of endothelial function in CAH *in vivo*

The pivotal role of the endothelium in vascular-related diseases has driven increased scientific attention in examining the endothelial function as a tool for screening, as well as for monitoring the course of the diseases and evaluating treatment outcomes (75). Traditionally, endothelial function is evaluated through endothelium-dependent vasomotion, which can be measured in either the coronary or peripheral circulation. While invasive angiography is still considered the gold standard for measuring coronary endothelial function (76), there is no agreement on the gold standard for the measurement of peripheral endothelial function (77).

Well-established non-invasive techniques for evaluating peripheral endothelial function include strain-gauge venous occlusion forearm plethysmography (78, 79), flow-mediated dilation (FMD) (79, 80), peripheral arterial tonometry (PAT) (79), and laser Doppler flowmetry (79). Less conventional non-invasive techniques include pulse wave velocity (PWV) (81) and indirect endothelial assessment via intima-media thickness (IMT) measurement (82) (**Table 1**). Unlike invasive techniques, which are associated with risks such as vascular injury, infection, and procedural complications, non-invasive methods are inherently safer, cost-efficient, logistically simpler, and well-suited for implementation in both clinical practice and large-scale epidemiological studies. According to several studies, non-invasive assessment of peripheral vascular function may be useful in identifying patients at risk for cardiac adverse events, including cardiac death, myocardial infarction, revascularization, or hospitalization for cardiac causes (83).

**Table 1:** Non-invasive methods of assessment of endothelial function.

Method	Technique	Advantages	Limitations
FMD	Measures endothelium-dependent vasodilation by assessing changes in the diameter of the brachial artery in response to increased blood flow after occlusion.	Non-invasive, cost-efficient, and widely accessible method. It utilizes validated digital software for accuracy and is clinically relevant in detecting vascular dysfunction.	Requires expertise and is sensitive to variations in protocols. External factors influence results, introducing confounding variables. Diagnostic specificity is limited in diseases with overlapping mechanisms.
PAT	Measures vascular response through pulsatile arterial volume changes in the finger during reactive hyperemia, providing an RHI.	Non-invasive, operator-independent, quick training for operators, and automated results.	Primarily reflects microvascular rather than macrovascular function. RHI cutoff values may not be reliable in younger populations due to limited post-occlusion arterial dilation.
PWV	Measures arterial stiffness by assessing the velocity of blood pressure waves between two arterial sites (e.g., carotid-femoral or brachial-ankle).	Simple, non-invasive, cost-effective, and reproducible. Considered the gold standard for assessing arterial stiffness. Provides insights into both central and peripheral arterial stiffness.	Does not directly measure endothelial function but arterial stiffness. Influenced by confounding factors such as blood pressure and arterial wall properties. Vasodilators are required to isolate the endothelial contribution to measurements.
IMT	Measures the thickness of arterial walls using ultrasound, commonly in the carotid arteries, to assess subclinical atherosclerosis and vascular health.	Non-invasive and well-established. Can track disease progression over time. Elevated IMT is a recognized marker of atherosclerosis and endothelial dysfunction.	Does not provide a direct functional assessment of the endothelium. Results may vary with operator expertise and differences in measurement protocols.

FMD, flow-mediated dilation; PAT, peripheral arterial tonometry; RHI, reactive hyperemia index; PWV, Pulse Wave Velocity; IMT, intima-media thickness.

### 4.1 Flow-mediated dilatation

One of the most used techniques to study endothelial function *in vivo* is FMD, which is assessed using ultrasound to measure changes in the diameter of the brachial artery in response to increased blood flow following a period of vascular occlusion created by a blood pressure cuff. This response is highly dependent on the availability of NO. Endothelial dysfunction is indicated by decreased vasodilation, as shown by lower FMD in the brachial artery.

The benefits of the FMD technique encompass cost-efficiency, non-invasiveness, accessibility, and the use of validated digital software for automated analyses. However, achieving optimal examination is technically demanding, and variations in techniques and protocols can impact the consistency and reliability of results. Moreover, factors such as diet, coffee consumption, medication, vitamins, physical activity, tobacco use, air temperature, and the menstrual cycle can influence a patient’s FMD, potentially introducing confounding variables into the study outcomes (28). Nonetheless, the brachial FMD method provides a validated, non-invasive evaluation of endothelial function (76).

Wierzbicka-Chmiel et al. (84) reported that 19 patients with CAH had decreased mean FMD compared with the control group. Farghaly et al. (16) showed that FMD was impaired in 40 patients with CAH and associated with elevated levels of neopterin and high sensitivity C-reactive protein (hs-CRP), the markers of vascular inflammation. According to Harrington et al. (85), impaired FMD was observed in a group of 14 adolescents with CAH, similar to obese control subjects. Given the limited number of studies assessing FMD in CAH, it is noteworthy that impaired FMD is a well-recognized indicator of endothelial dysfunction in other conditions associated with increased cardiovascular risk, such as type 2 diabetes (86–88), PCOS (89–91), obesity (92–94), heart failure (95), and peripheral artery disease (96).

### 4.2 Peripheral arterial tonometry

The PAT method, which is used in the EndoPAT device (Itamar Medical Inc., Caesarea, Israel), has emerged as a newer than FMD, non-invasive technology for measuring endothelial dysfunction. The device uses non-invasive pneumatic probes placed on both index fingers to measure pulsatile arterial volume changes at rest and during reactive hyperemia, which occurs in response to increased shear stress. A blood pressure cuff is placed over the brachial artery and inflated to occlude blood flow, and the response after deflation is recorded. The pulse wave amplitude (PWA) is measured, and the reactive hyperemia index (RHI) result is automatically calculated. The RHI is calculated as the ratio of the average PWA during the reactive phase to the average amplitude measured during the stabilization period. A suggested RHI threshold for indicating endothelial dysfunction is <1.67 (97–99). To adjust for systemic changes, this ratio is normalized using the concurrent signal from the contralateral finger. The EndoPAT device also assesses the peripheral augmentation index (AI), which measures arterial stiffness, calculated based on PWA.

The main advantages of PAT technology include its easy accessibility, operator independence, automated calculation, and control by the contralateral arm (83). PAT operators can be trained in a relatively short amount of time and do not require specialized certification. A major limitation is the unclear understanding of its pathophysiological basis (100). Unlike FMD, which evaluates macrovascular dilation, PAT assesses microvessel dilation. The endpoint measured by PAT, PWA, is thought to reflect arterial distensibility and venous capacitance in the digital vasculature, suggesting that changes in PWA may indicate vascular function. However, the structure of the digital vasculature is complex, comprising both nutritive vessels and arteriovenous anastomoses. The sympathetic nervous system primarily regulates the resting vascular tone in these vessels, with NO playing a minimal role (101). There are also concerns regarding the cutoff point for RHI, particularly in relation to age. Jujic et al. (102) postulated that an RHI result under 1.67, an early marker of endothelial dysfunction, may not be a suitable measure of endothelial function in individuals under 30 years of age. Their findings suggest that low RHI in young, healthy individuals may not necessarily indicate true endothelial dysfunction, but rather be an artefact of the limited ability of healthy arteries to dilate post-occlusion (102).

Available data on the positive predictive value (PPV) and negative predictive value (NPV) of the EndoPAT test are heterogeneous and vary depending on the studied population and clinical context. For instance, in the evaluation of erectile dysfunction, the PPV was relatively low at 43%, whereas the NPV reached 90%, indicating a greater utility in excluding rather than confirming organic endothelial dysfunction (103). In contrast, when EndoPAT was compared to the acetylcholine provocation test—the gold standard for diagnosing coronary endothelial dysfunction—the sensitivity was reported at 80% and specificity at 85%, supporting its potential role in identifying coronary artery spasm (99). These differences highlight the importance of interpreting EndoPAT results within the appropriate clinical framework.

Despite several limitations, numerous studies in both adult and pediatric literature reveal PAT’s satisfactory reproducibility and reliability (104–106). To date, no study utilizing PAT technology in individuals with CAH has been published. However, reduced RHI values have been reported in patients with other conditions associated with increased cardiovascular risk, including coronary artery disease (107), type 2 diabetes (108, 109), and metabolic syndrome (110). In contrast, the levels of RHI were consistent with preserved endothelial function in both groups of patients with PCOS, whether or not they had non-alcoholic fatty liver disease (NAFLD) (111).

### 4.3 Pulse wave velocity

PWV is the proposed gold standard for arterial stiffness and an indicator of early atherosclerosis (112), however, this technique can also be applied to studies of endothelial function (81). The predictive value of PWV for the occurrence of cardiovascular diseases has been demonstrated in both the general population and patients with various clinical conditions, including hypertension (113, 114), type 2 diabetes (115), end-stage renal disease (116), stroke (117), and coronary artery disease (118).

Measuring PWV involves the delay in the peak of the peripheral pulse wave, typically in relation to the QRS complex recorded simultaneously using electrocardiography. Increased vessel stiffness results in faster pulse wave propagation. The delay is determined by measurements taken at two different body sites, such as the carotid and femoral arteries (carotid-femoral PWV) or brachial and ankle arteries (brachial-ankle PWV). These two PWVs are most widely used in clinical and research fields. By selecting specific pulse wave recording points, it is possible to assess both central arterial stiffness and peripheral stiffness, independent of aortic condition. In endothelial studies, PWV measurements rely on the assumption that administering vasodilators isolates the contribution of vascular tone (119). PWV measurement is clinically beneficial due to its simplicity, non-invasive nature, cost-effectiveness, and reproducibility (120). Currently, no data are available on the PPV or NPV of PWV measurement in the assessment of endothelial function.

Costa et al. (121) did not observe any significant differences in PWV between 47 women with NCCAH and controls, nor across different therapy groups (dexamethasone vs. contraceptive pills). Similarly, Rosenbaum et al. (122) reported no significant differences in PWV values between 84 patients with CAH (both CCAH and NCCAH) and controls.

However, outside the CAH population, many studies have recently revealed an association between increased PWV and coronary atherosclerosis (123–125). In patients with PCOS, PWV measured at the brachial artery was found to be significantly elevated, although aortic PWV did not differ between the PCOS and control groups (126). Moreover, Wang et al. (127) reported that brachial-ankle PWV was associated with metabolic syndrome and increased progressively with the number of metabolic syndrome components in the general population.

### 4.4 Intima-media thickness

IMT is a measurement of the thickness of artery walls by ultrasound to detect the presence or track the progression of cardiovascular disease. In clinical practice, the IMT measurement is most commonly performed in the carotid arteries. A common carotid intima-media thickness (cIMT) measurement greater than 0.9 mm has been considered a significant factor influencing cardiovascular prognosis (128). Since increased IMT is a widely recognized sign of endothelial impairment (82) and a recognized indicator of atherosclerosis (129), which is closely linked to endothelial dysfunction, it can be used as an indirect measure of endothelial condition. Several studies have linked endothelial dysfunction with cIMT in patients with established atherosclerosis or coronary artery disease (130, 131). Consistently, endothelial dysfunction, defined as endothelium-dependent vasodilation (EDV) ≤4.5%, has been associated with a sensitivity of 71%, a specificity of 81%, and a PPV of 95% for the presence of coronary artery disease, further supporting the role of early vascular changes as markers of impaired endothelial function (130).

The exploration of IMT in relation to CAH has been the subject of multiple studies (60, 122, 132–138), with results varying significantly. Most of the studies (133–139) have reported notable differences in IMT between patients with CAH and controls, however, some research has shown normal IMT values, similar to those of control groups (60). The increase in cIMT is more pronounced in adults compared to youth (56), however, only three studies have examined adults (122, 133, 140), while the rest have focused on pediatric and adolescent populations (60, 134–140). Rosenbaum et al. (122) observed no significant difference between cIMT values in 84 adult patients with CAH and controls. Kim et al. (140) found that cIMT was associated with elevated androgen levels in 20 adolescents and young adults with CAH, with a loss of sex differences observed in female patients with excess androgen exposure. However, the subjects with CAH did not have significantly different cIMT values compared to controls. They also found that cIMT was significantly greater in obese than in non-obese individuals with CAH (140). Amr et al. (137) reported significantly higher CIMT in 32 children with CAH, without differences between the SV and SW forms of CCAH. Wasniewska et al. (138) observed increased IMT at different sites in 18 adolescents with CAH, with no differences between CCAH and NCCAH. Rodrigues et al. (136) has found remarkably higher values of cIMT in 40 patients with CAH, with no notable differences between those of normal weight and those who were overweight. Özdemir et al. (139) observed that 25 children with CAH exhibited higher IMT and decreased distensibility of the aorta and carotid arteries compared to control subjects, indicating the potential for early subclinical atherosclerosis. Akyürek et al. (134) observed that cIMT was higher in hypertensive compared to normotensive patients with CAH. Metwalley et al. (135) identified a significant correlation between cIMT and markers of disease management, including treatment duration and levels of 17-OHP and testosterone, indicating that elevated androgen levels may contribute to an increased risk of vascular dysfunction. Notably, cIMT showed no correlation with the hydrocortisone dose equivalent (135).

## 5 Novel biomarkers of endothelial function in CAH

A broad spectrum of potential biomarkers linked to endothelial function has been identified in reviews addressing cardiovascular diseases (11, 141, 142), renal diseases (143), and peripheral vascular diseases (144, 145). The most extensively studied include endothelial progenitor cells (146, 147), endothelial microparticles (148), microRNAs (149, 150), and adhesion molecules such as P-selectin (151), E-selectin (152), ICAM-1 (153), and VCAM-1 (153), along with molecules involved in the coagulation pathway, particularly von Willebrand factor (154). Although the levels of these well-characterized biomarkers have not yet been investigated in individuals with CAH, some data are available on less extensively studied or newly identified biomarkers, including neopterin (155), osteoprotegerin (132, 156), fetuin A (157), homocysteine (158, 159), leptin (42, 57, 160–163), adiponectin (60, 164), C-reactive protein (CRP) (55, 165), hsCRP (38, 57, 135), interleukin 6 (IL-6) (55, 57, 121, 166), and circulating endothelial cells (CECs) (135) (**Table 2**).

**Table 2:** Biomarkers that play a significant role in endothelial dysfunction.

Marker	Description	Role in the endothelium	Role in CAH
Neopterin	A metabolite of GTP, secreted by activated macrophages in response to interferon-γ.	Promotes adhesion molecule expression (ICAM-1, VCAM-1) and contributes to oxidative stress, impairing endothelial NO bioavailability.	Elevated levels are associated with endothelial dysfunction, contributing to vascular inflammation and oxidative stress (16).
Osteoprotegerin	A protein involved in bone metabolism by inhibiting osteoclast activity and regulating RANK/RANKL signaling.	Induces the expression of endothelial adhesion molecules (VCAM-1, ICAM-1, E-selectin).	Linked to increased cardiovascular risk through inflammatory pathways. Data regarding the impact on bone mineral metabolism remain inconclusive (132, 156, 167).
Fetuin-A	A glycoprotein expressed in the liver that modulates insulin resistance by binding to the insulin receptor and inhibits vascular calcification.	Prevents vascular calcification by inhibiting calcium-phosphate precipitation but contributes to endothelial dysfunction through insulin resistance and inflammation.	Elevated levels are associated with androgen excess and insulin resistance, potentially exacerbating metabolic complications (154).
Homocysteine	A sulfur-containing amino acid involved in methionine and cysteine metabolism.	Increases ROS production, impairs NO synthesis, and triggers pro-inflammatory cytokine release, exacerbating endothelial activation and dysfunction.	Elevated levels are associated with increased cIMT, higher left ventricular mass index, and prolonged mitral deceleration time, indicating a potential risk for subclinical atherosclerosis and left ventricular dysfunction in children (159). Conversely, lower concentrations may be linked to cardiovascular protection (158).
Leptin	A hormone secreted by adipocytes.	Promotes endothelial dysfunction through oxidative stress, inflammation, and vascular smooth muscle proliferation.	Elevated levels are linked to metabolic disturbances, including higher BMI, insulin resistance, obesity, and body fat percentage. (32, 38, 60, 160).
Adiponectin	An adipokine with anti-inflammatory, anti-fibrotic, and antioxidant properties that regulates glucose and lipid metabolism and insulin sensitivity.	Reduces inflammation, increasing NO bioavailability, and preventing oxidative stress.	Increased levels may act as a compensatory mechanism to mitigate metabolic and vascular risks in pediatric patients (60).
PAI-1	A protein that regulates fibrinolysis by inhibiting tissue and urokinase plasminogen activators.	Inhibits fibrinolysis, promotes thrombosis, and enhances vascular inflammation.	PAI-1 levels positively correlate with visceral and subcutaneous adipose tissue (38).
CRP	An acute-phase protein produced by the liver in response to inflammation.	Induces pro-inflammatory signaling in endothelial cells, upregulates adhesion molecules (ICAM-1, VCAM-1), and decreases NO production, worsening endothelial dysfunction.	Elevated CRP and hsCRP levels, especially in poorly controlled CAH, indicate systemic inflammation and increased cardiovascular risk (16). A positive correlation between hsCRP levels and the amount of visceral and subcutaneous adipose tissue in adolescents and young adults has been observed (38).
IL-6	A pleiotropic cytokine involved in immune regulation, inflammation, and vascular function, influencing endothelial NO bioavailability and oxidative stress.	Increases endothelial permeability, upregulates adhesion molecules, reduces NO bioavailability, and promotes ROS production.	Elevated levels may be linked to dexamethasone therapy and inflammation (121).
CECs	Mature, non-hematopoietic cells shed into the bloodstream following vascular injury or during normal endothelial turnover.	CECs are sensitive biomarkers of endothelial damage.	Higher levels may be linked to subclinical atherosclerosis and low-grade vascular inflammation in children with CAH (135).

GTP, guanosine triphosphate; PAI-1, plasminogen activation inhibitor 1; ROS, reactive oxygen species; cIMT, carotid intima-media thickness; CRP, C-reactive protein; hs-CRP, high sensitivity C-reactive protein; IL-6, interleukin 6; CECs, circulating endothelial cells.

Although biomarkers hold significant promise, standardized chemistry tests and protocols for evaluating endothelial damage are not yet available and are currently limited to clinical research applications. It has been suggested that the most effective approach for assessing endothelial function could involve combining tests for circulating endothelial biomarkers with vasomotor response assessments (10). More recently, researchers have been exploring a multibiomarker strategy that integrates both traditional and novel circulating markers (168), offering a potentially more robust tool for cardiovascular risk stratification and therapy monitoring.

### 5.1 Neopterin

Neopterin, a metabolite of guanosine triphosphate (GTP), is synthesized by activated macrophages in response to stimulation by γ-interferon secreted by activated T-lymphocytes (169). Neopterin promotes atherothrombosis by increasing tissue factor-mRNA transcription and the expression of adhesion molecules ICAM-1 and VCAM-1 (170), however, it also suppresses TNF-α-induced expression of MCP-1, ICAM-1, and VCAM-1, reducing adhesion to endothelial cells, and inhibiting macrophage foam cell formation and smooth muscle cell proliferation (171). Neopterin levels correlate with ROS production and its toxic effects (172), making neopterin an indirect marker of oxidative stress during cell-mediated immune responses. Its vascular role in atherosclerotic processes, either beneficial or deleterious, is still under investigation.

Farghaly et al. (16) reported that patients with CAH had higher neopterin levels compared to healthy controls. These elevated neopterin levels were significantly associated with endothelial dysfunction, as demonstrated by brachial artery FMD measurements (16). Given the limited research on neopterin in CAH, it is relevant to note that increased plasma neopterin levels have also been observed in various other conditions characterized by vascular or systemic inflammation, including atherosclerosis (173, 174), coronary artery disease (175), hypertension (176), and ischemic stroke (177).

### 5.2 Osteoprotegerin

Osteoprotegerin is a member of the tumor necrosis factor (TNF) receptor superfamily, playing a crucial role in regulating bone metabolism (178). It is a glycoprotein that prevents the differentiation and activity of osteoclasts by binding to the receptor activator of nuclear factor-kappa B ligand (RANKL), thereby inhibiting RANKL from interacting with its receptor, RANK. This blockage of the RANK/RANKL pathway leads to decreased osteoclast formation and reduced survival and activation of mature osteoclasts (179, 180). Through these mechanisms, osteoprotegerin contributes to preserving the balance between bone resorption and formation (179). Additionally, TNF-related apoptosis-inducing ligand (TRAIL), another member of the TNF superfamily, also interacts with osteoprotegerin (181).

Apart from its role as a regulator of bone metabolism, osteoprotegerin has recently emerged as a significant factor in in the pathogenesis of atherosclerosis and cardiovascular diseases (182, 183), amplifying the adverse effects of inflammation and several traditional risk factors such as hyperlipidemia, type 2 diabetes, and hypertension (184). Furthermore, genetic studies have shown associations of osteoprotegerin gene polymorphisms with cardiovascular disease (185, 186). Notably, osteoprotegerin binds directly to RANKL, interfering with its interaction with the RANK receptor on the endothelium and thereby regulating vascular calcification (187, 188). It can act as a receptor for TRAIL, inhibiting the effects of TRAIL on the up-regulation of eNOS and down-regulation of ROS production. In addition, osteoprotegerin also activates the renin-angiotensin system and induces vascular endothelial growth factors, leading to inflammatory and fibrotic processes (189, 190). It has also been demonstrated to induce the expression of VCAM-1, ICAM-1, and E-selectin on endothelial cells, promoting leukocyte adhesion, which is an early step in endothelial cell dysfunction (191).

Although the link between osteoprotegerin concentration and cardiovascular risk in patients with CAH has not been evaluated yet, evidence suggests an association between osteoprotegerin and bone metabolism in people with CAH (132, 156, 167). It was found that osteoprotegerin levels were significantly higher in children with CAH compared to controls, indicating a compensatory mechanism against increased bone resorption in CAH (156). In contrast, another study showed that children with CAH had significantly lower serum osteoprotegerin levels (132), similar to a case report in which a lower serum osteoprotegerin level was found in a child with CAH (167).

Given the limited and inconsistent data on osteoprotegerin in CAH, insights from other conditions characterized by endothelial dysfunction may offer a broader perspective on the potential vascular implications of altered osteoprotegerin levels. Several studies have demonstrated a significant association between osteoprotegerin concentrations and endothelial dysfunction, particularly in patients with hyperuricemia (192), type 2 diabetes mellitus (193), and HIV infection (194). However, studies examining individuals with diabetes (195) or PCOS (196), have reported no correlation between osteoprotegerin levels and alterations in endothelial function.

### 5.3 Fetuin-A

Fetuin-A is a heterodimer plasma glycoprotein of the cystatin superfamily of protease inhibitors, predominantly expressed in the liver (197). It contributes to insulin resistance by binding to the tandem fibronectin type 3 domains in the extracellular β-subunit of the insulin receptor, inhibiting its activity in peripheral tissues. This binding occurs away from the α-subunits, where insulin binds, with both insulin and fetuin-A targeting the receptor’s ectodomain; however, while insulin activates the receptor’s intrinsic tyrosine kinase activity, fetuin-A deactivates it (198).

Increased fetuin-A levels are associated with various metabolic health factors such as insulin sensitivity (199), glucose tolerance (200), lipid concentrations (201), and inflammatory cytokine levels (202). However, fetuin-A plays an important role in preventing cardiovascular calcification by binding to small calcium-phosphate complexes, preventing their expansion, aggregation, and precipitation (203). It also promotes phagocytosis of extracellular vesicles and apoptotic cells by vascular smooth muscle cells and macrophages, which helps reduce both apoptosis and calcification in conditions with elevated extracellular mineral ion concentration in tissues (204).

Kurnaz et al. (157) observed elevated levels of fetuin-A in 56 patients with CCAH, compared to a control group, along with increased insulin levels and disrupted insulin signaling. Furthermore, the high levels of fetuin-A and insulin substances showed a positive correlation with free and total testosterone. The authors postulated that these findings could be linked to androgen excess and prolonged or high-dose glucocorticoid therapy in people with CAH (157). They suggested that the presence of androgen receptors in liver cells may allow excess androgens to stimulate the overproduction of fetuin-A and insulin, potentially contributing to insulin resistance (157, 205).

The potential involvement of fetuin-A in the metabolic disturbances observed in CAH is further supported by studies in other conditions characterized by insulin resistance and increased cardiometabolic risk. Higher circulating levels of fetuin-A have been linked to obesity (206) and its related conditions, including type 2 diabetes (207), metabolic syndrome (201, 206), PCOS (208) (209), and NAFLD (200). Liu et al. (208) observed that patients with PCOS had higher circulating fetuin-A levels compared to healthy women. Furthermore, higher fetuin-A levels correlated positively with BMI, waist-to-hip ratio, LDL cholesterol and triglyceride concentrations, and other indicators (208). Enli et al. (209) also observed higher fetuin-A levels in patients with PCOS, which correlated positively with insulin, the Homeostasis Model Assessment of Insulin Resistance (HOMA-IR), and the free androgen index.

### 5.4 Homocysteine

Homocysteine, an amino acid containing a sulfhydryl group, is an intermediate product in the metabolism of methionine and cysteine that affects many cellular biological processes, such as cellular methylation status, cell metabolism, and cell injury (210). Increased concentration of fasting plasma homocysteine (higher than 15 μmol/L) is defined as hyperhomocysteinemia (211), and is associated with various neurodegenerative, metabolic, and cardiovascular disorders (83, 212–218).

Hyperhomocysteinemia contributes to endothelial dysfunction through several mechanisms. First, it stimulates the overproduction of ROS, leading to oxidative stress, which impairs the bioavailability of NO and results in impaired blood vessel relaxation (219). Second, high homocysteine levels induce the release of pro-inflammatory cytokines and other inflammatory mediators, which contribute to the activation of endothelial cells and promote vascular inflammation (220). Lastly, hyperhomocysteine can impair the activity of eNOS (221).

Metwalley et al. (159) observed elevated homocysteine levels in 36 children with CAH, particularly among those with poor disease control. This elevation correlated with increased cIMT, left ventricular mass index, and mitral deceleration time, indicating a potential risk for subclinical atherosclerosis and left ventricular dysfunction (159). Krysiak et al. (222) found that the mean homocysteine concentration was significantly higher in patients with NCCAH compared to the control group. This difference remained significant after adjusting for age and BMI. In contrast, Falhammar et al. (158) reported lower homocysteine levels in male patients with CAH under 30 years of age compared to controls, suggesting a potential association with cardiovascular protection. However, it was proposed that the differing results could be attributed to variations in age, sample size, ethnicity, dietary habits, genetic factors, research methods, and the different steroid treatments (223). Bayraktar et al. found no statistically significant difference in mean homocysteine concentrations between 50 patients with CAH and the control group.

Beyond CAH, hyperhomocysteinemia has been widely studied in other conditions characterized by metabolic dysfunction and increased cardiovascular risk. Elevated serum homocysteine concentrations have been demonstrated in women with PCOS (224–226), in obese and overweight patients with hypertension, and in normotensive obese individuals (227). Furthermore, a study by Vaya et al. (228) reported that homocysteine levels in morbidly obese patients were associated with increased waist circumference and insulin resistance.

### 5.5 Leptin

Leptin is a pleiotropic hormone secreted by adipocytes that plays a role in various biological processes, such as angiogenesis, vascular function, inflammatory response, bone homeostasis, and reproduction (229). Increased serum leptin levels are directly associated with higher adipose tissue mass and are a significant contributor to obesity and its metabolic complications.

The reports on the role of leptin are contradictory in CAH. A study by Charmandari et al. (42) demonstrated significantly elevated leptin concentrations in 18 young patients (2–12 years old) with CCAH, compared to controls, regardless of BMI and sex. Leptin levels were inversely correlated with epinephrine and free metanephrine concentrations, suggesting a reduced inhibitory effect of catecholamines on leptin secretion (42). Additionally, the group with CAH exhibited a loss of gender dimorphism in leptin concentrations, possibly due to androgen excess in female patients (42). Increased leptin levels and strong correlations between leptin, obesity (32, 38, 60), and HOMA-IR (60) have been observed in studies including both patients with CCAH and NCCAH. Interestingly, Borges et al. (160) demonstrated significantly elevated leptin secretion, along with higher BMI and body fat percentage, exclusively in male patients with CAH compared to controls, while no differences were observed in female patients. In contrast, several studies have reported leptin concentrations in patients with CAH to be comparable to those of controls, despite higher BMI and body fat (57, 161–163). However, Volkl et al. (161) observed significantly lower concentrations of soluble leptin receptors in 51 individuals with CAH compared to the control group, suggesting an increased level of free (unbound) serum leptin.

In addition to findings in CAH, numerous studies have investigated the role of hyperleptinemia in cardiovascular diseases, including congestive heart failure, myocardial infarction, hypertension, and coronary artery disease (230, 231), highlighting its potential contribution to vascular dysfunction. It has been found that elevated plasma leptin levels are associated with coronary artery calcification (232) and with higher serum cholesterol, triglycerides, and CRP levels in patients with coronary artery disease (233). In contrast to these findings, several other studies have suggested that leptin may exert protective effects on blood vessels in obese individuals (234).

### 5.6 Adiponectin

Adiponectin, an adipokine secreted by adipocytes, is a well-known homeostatic factor that regulates glucose levels, lipid metabolism, and insulin sensitivity through its anti-inflammatory, anti-fibrotic, and antioxidant effects (235). In addition to adipocytes, adiponectin is also expressed in other tissues, including liver parenchyma cells (236), myocytes (237), and epithelial cells (238). The effects of adiponectin are primarily mediated through adiponectin receptors (AdipoR1 and AdipoR2) (239), and also via T-cadherin receptor (240). Adiponectin acts directly on the liver, skeletal muscle (239), and vasculature, including endothelial cells (241), smooth muscle cells (240), and pericytes (242).

Increasing evidence suggests that adiponectin plays a protective role in the cardiovascular system; it was found that adiponectin is inversely correlated with an increased cardiovascular risk, and hypo-adiponectinemia is associated with coronary artery disease and hypertension (243), left ventricular hypertrophy (244), and a greater risk of myocardial infarction (245). Furthermore, adiponectin is linked to the regulation of energy balance, enhanced angiogenesis, anti-inflammatory responses, antiapoptotic effects, and the prevention of interstitial fibrosis (246, 247). It protects the myocardium against oxidative stress and damage from ischemia/reperfusion, and it reduces cardiac remodeling caused by pressure overload or following a myocardial infarction (248, 249).

Adiponectin levels are known to be decreased in conditions such as obesity, insulin resistance, and type 2 diabetes (250), and the administration of glucocorticoids and androgens has been shown to reduce adiponectin levels (251, 252). However, the role of adiponectin in the cardiometabolic risk of patients with CAH remains unclear. Völkl et al. (164) reported higher adiponectin concentrations in 51 individuals with CAH compared to controls, regardless of sex, with no observed changes in serum leptin or the adiponectin/leptin ratio. Adiponectin levels were negatively correlated with BMI, serum dehydroepiandrosterone, and testosterone, but no clear relationship was found with hydrocortisone or fludrocortisone dosage (164). Similarly, Mooij et al. (60) observed a trend towards elevated adiponectin levels in 27 patients with CAH, compared to controls, which may suggest that adiponectin has a protective role in these patients.

### 5.7 Plasminogen activation inhibitor 1

Plasminogen activation inhibitor 1 (PAI-1) is the primary physiological inhibitor of urokinase plasminogen activator (u-PA) and tissue-plasminogen activator (tPA) and is a member of the serpin superfamily (serine proteinase inhibitors). Its most important function is to regulate plasminogen activator activity, thereby controlling plasmin formation. Consequently, PAI-1 plays a vital role in systemic homeostasis, contributing to the balance of the coagulation and fibrinolytic processes (253).

Data on PAI-1 levels in individuals with CAH and their relationship with cardiovascular and metabolic disorders are limited. Mooij et al. (55) found no significant differences in PAI-1 levels between patients with CAH and controls. Kim et al. (38) observed a positive correlation between PAI-1 levels and the amount of visceral and subcutaneous adipose tissue, both of which were higher in patients with CAH compared to controls. These findings suggest a potential link between PAI-1 and adverse metabolic profiles in CAH, although further research is needed to clarify its role in this population.

Evidence from other conditions shows that elevated PAI-1 levels are linked to the development of cardiovascular and metabolic disturbances, including atherosclerosis (254), type 2 diabetes, obesity, metabolic syndrome (255–257), and PCOS (258, 259). Moreover, increased levels of glucose (260), insulin (261) and its precursors (262), and free fatty acids (263) have been demonstrated to promote PAI-1 expression or to decrease the degradation rate of PAI-1 mRNA (264), further contributing to a prothrombotic and hypofibrinolytic state.

### 5.8 C-reactive protein

CRP is produced by the liver in response to inflammation and is regulated, in particular, by IL-6 and TNF-α. It is considered a prototypic marker of inflammation. CRP contributes to atherosclerosis by inducing pro-inflammatory signaling in monocytes and increasing cytokine production. In endothelial cells, it upregulates cell adhesion molecules, MCP-1, endothelin-1, and PAI-1, while decreasing prostacyclin release and tPA activity (265). Additionally, CRP has been shown to reduce eNOS activity and lower NO levels (165).

HsCRP is measured using high-sensitivity tests, which enable the detection of very low concentrations, making it valuable for assessing cardiovascular risk in seemingly healthy individuals. A body of evidence supports the utility of this marker in evaluating cardiovascular risk [reviewed by Ridker et al. (266)]. Nevertheless, although CRP has shown consistency as a cardiovascular risk biomarker across large prospective studies, with relative risk ratios approaching those of classical cardiovascular risk factors, its contribution to the current evaluation model of endothelial dysfunction remains minor (267).

Despite the clear impact of inflammation on the development of endothelial dysfunction, data on inflammatory markers in CAH are scarce. In a study of 40 adult patients with CAH, hsCRP levels were shown to be markedly elevated compared to controls, and they were even more pronounced in patients with poorly controlled disease (16). Similar findings have been reported in 32 children (135) and 21 adolescents (57) with CAH. Kim et al. (38) observed a positive correlation between hsCRP levels and the amount of visceral and subcutaneous adipose tissue in 28 adolescents and young adults with CAH. However, a Dutch study involving a cohort of 27 adult patients with CAH showed no differences in the levels of CRP or other inflammatory markers, such as IL-6 and IL-18, compared to healthy controls (55).

### 5.9 Interleukin 6

IL-6 is a pleiotropic cytokine involved in the regulation of immune responses by recruiting macrophages and lymphocytes to sites of injury or infection, acting as both a pro-inflammatory and anti-inflammatory agent. It also plays a role in a plethora of other functions, ranging from shaping the blood-brain barrier permeability to synovial inflammation, hematopoiesis, embryonic development, and vascular permeability (268–270). As IL-6 is involved in the development of autoimmunity and plays a crucial role in sepsis, targeting the IL-6 axis is an approved pharmacological option in a number of autoimmune disorders. IL-6 is synthesized locally at the inflammation site from where it is transported via the bloodstream and exerts its action on the liver, immune cells, and endothelium [reviewed by Tanaka et al. (271)]. In hepatocytes, its signaling leads to the synthesis of, in particular, CRP, serum amyloid A, antitrypsin, hepcidin, fibrinogen, thrombopoietin, and complement cascade elements (272). In the vessels, IL-6 not only acts directly on endothelial cells to increase the production of cytokines and chemokines, thereby activating the coagulation cascade, but also enhances vascular permeability by inducing VE-cadherin disassembly and promotes clot formation by stimulating tissue factor expression on monocytes (270). Moreover, IL-6 directly affects eNOS activity and increases vascular superoxide, which rapidly inactivates NO, thereby reducing NO bioavailability (273). IL-6 contributes to atherosclerosis by upregulating angiotensin II type 1 receptor expression (274), while increased binding of angiotensin II in turn increases IL-6 signaling (270, 275).

The unique role of IL-6 in shaping cardiovascular risk is demonstrated by experiments in mice with conditional overexpression of IL-6. These experiments demonstrated significantly impaired endothelium-dependent aortic relaxation, ROS formation in the aorta, and vascular dysfunction, all of which led to a markedly decreased survival rate (276). Due to its action on endothelial cells, IL-6 is considered both a marker of cardiovascular risk and a predictor of long-term cardiovascular mortality (277, 278).

According to Ariyawatkul et al. (57), IL-6 was unaltered in the cohort of 21 adolescents with CAH when compared to their healthy peers. Similarly, Delai et al. (166) found no significant differences in IL-6 levels between 31 patients with NCCAH and controls, suggesting that IL-6 may not directly contribute to insulin resistance in this population. Costa et al. (121) reported higher serum IL-6 levels in a group of 28 women with NCCAH treated with dexamethasone compared to 19 women treated with contraceptive pills. However, no statistically significant difference was found in IL-6 serum levels when comparing either group to the control group (121).

### 5.10 Circulating endothelial cells

CECs are mature, non-hematopoietic cells shed into the bloodstream following vascular injury or during normal endothelial turnover (279). Under physiological conditions, they are present in minimal numbers; however, elevated CEC counts have been documented across a variety of cardiovascular, oncological, and inflammatory diseases, underscoring their potential as sensitive biomarkers of endothelial damage. The enumeration and phenotyping of CECs—primarily via advanced multiparameter flow cytometry protocols—offer a non-invasive “liquid biopsy” approach to assess vascular health​ (280, 281). Unlike traditional endothelial markers, such as soluble adhesion molecules or von Willebrand factor, CECs directly reflect structural endothelial injury rather than secondary activation processes (282).

In a case-control study by Metwalley et al. (135), 32 children with CAH exhibited significantly higher CEC counts compared to healthy controls, alongside increased CA-IMT and elevated hsCRP levels. These findings support the presence of subclinical atherosclerosis and low-grade vascular inflammation in CAH. Moreover, CEC levels correlated positively with markers of androgen excess (testosterone, 17-OHP) and with measures of cardiac dysfunction, suggesting that chronic hormonal imbalance and insufficient disease control may exacerbate endothelial damage (135)​​.

The observed increase in CECs in CAH is consistent with findings from other clinical contexts, where elevated CEC counts have been established as markers of endothelial injury and disease severity. Increased CEC levels have been reported in cardiovascular conditions such as acute coronary syndromes (283), heart failure (284), and deep vein thrombosis (285). Similarly, increased CEC counts have been documented in patients with solid tumors (e.g., colorectal, breast, small-cell lung cancer) (286–288) and hematological malignancies such as multiple myeloma (289, 290), correlating with tumor progression and response to therapy​. Inflammatory and autoimmune diseases, including systemic lupus erythematosus (291, 292) and small-vessel vasculitis (293), are also associated with higher CEC counts, often indicating active disease and worse prognosis.

## 6 Omics-based tools in understanding endothelial dysfunctions

In parallel with classical biomarkers, omics-based technologies, including genomics, proteomics, and metabolomics, are emerging as powerful tools for the discovery of novel biomarkers of endothelial dysfunction, particularly relevant in CAH. Genomic studies have identified polymorphisms in genes regulating vascular tone, inflammation, and oxidative stress [reviewed by Kim et al. (294)], which may modify individual cardiovascular risk in CAH. Proteomic analyses facilitate the identification of endothelial-derived proteins associated with oxidative stress and vascular remodeling (295, 296), whereas metabolomics provides insights into metabolic alterations affecting endothelial health, including disruptions in arginine metabolism and lipid oxidation (297).

Although omics technologies have demonstrated great promise in various cardiovascular and metabolic diseases, their application to the assessment of endothelial function in patients with CAH has not yet been explored. Despite their advantages, such approaches remain primarily within the domain of scientific research. Their clinical implementation is currently limited by factors such as high costs, technical complexity, and the need for specialized equipment and expertise, which restrict their use to selected research centers. Consequently, omics-derived biomarkers are not routinely used in clinical practice, and further research and validation in well-characterized CAH cohorts are necessary before broader application can be considered.

## 7 Therapeutic strategies to enhance cardiovascular health in CAH

Current glucocorticoid replacement in CAH remains non-physiological, often leading to androgen excess or glucocorticoid overtreatment. To address these issues, new approaches aiming to replicate circadian cortisol rhythms have been developed, including modified-release hydrocortisone (MR-HC, Efmody/Chronocort), immediate-release tablets with a sustained-release core (SR-HC, Plenadren), and continuous subcutaneous hydrocortisone infusion.

MR-HC improves biochemical control of 17OHP but has shown inconsistent effects on blood pressure, fat mass, and glucose metabolism; early morning cortisol peaks may transiently worsen insulin sensitivity (298–300). Interestingly, a 6-month MR-HC therapy in patients with SW-CAH resulted in a reduction of plasma renin activity and an increase in sodium levels, suggesting more effective mineralocorticoid action, likely due to decreased concentrations of 17OHP, a known mineralocorticoid receptor antagonist (298). SR-HC, although effective in primary and secondary adrenal insufficiency, appears suboptimal in CAH due to insufficient pre-awakening cortisol rise and weak androgen suppression (301, 302). Continuous hydrocortisone infusion more closely mimics physiological cortisol rhythms and enables dose reduction; however, data on its metabolic effects are limited, and its clinical use will likely remain restricted to highly selected patients due to its complexity (303). Emerging therapeutic options, including CRF-1 antagonists (crinecerfont and tildacerfont), have shown potential to reduce adrenocorticotropic hormone (ACTH) and androgen levels, enabling possible glucocorticoid dose reduction, although their long-term impact remains to be established (304–306).

In parallel, adjunctive therapies aiming to counteract androgen excess, such as oral contraceptives or spironolactone, play a role in the management of CAH, although they introduce additional metabolic and cardiovascular concerns requiring careful consideration (307). Combined hormonal contraceptives have been associated with an increased risk of venous thromboembolism and hypertension (308), however, they may assist in regulating menstrual cycles and reducing androgenic symptoms in individuals with CAH. Spironolactone, commonly used in women with androgen excess, improves cardiovascular outcomes in various cardiac conditions. Nevertheless, its cardiometabolic safety in the context of androgen excess remains unestablished, and its use in CAH may be limited due to the frequent coexistence of aldosterone deficiency (309, 310).

Given the elevated cardiometabolic risk in patients with CAH, especially those predisposed to developing diabetes, interventions commonly used in the general population are considered applicable, although evidence specific to CAH remains limited. Intensive lifestyle modifications—encompassing dietary improvements, increased physical activity, smoking cessation, and weight management—are recommended as first-line measures (311). Isolated case reports have demonstrated the beneficial effects of metformin (312, 313), topiramate (314), and bariatric surgery (315, 316) on weight reduction, visceral fat mass, and insulin sensitivity in patients with CAH. Newer antiobesity medications (liraglutide, semaglutide, tirzepatide, and naltrexone/bupropion) have not yet been studied in patients with CAH.

Statin therapy is considered safe and effective for lipid management in CAH, demonstrating significant reductions in total and LDL cholesterol (317, 318) and potential antiandrogenic effects (318). Despite theoretical concerns regarding statin- and glucocorticoid-induced myopathy (319, 320), no such cases have been reported in CAH, likely due to the use of moderate doses.

Management of hypertension in CAH should prioritize angiotensin-converting enzyme (ACE) inhibitors and angiotensin-receptor blockers (ARBs) due to their cardioprotective properties, beneficial effects on endothelial function, and ability to improve insulin sensitivity (321, 322). Thiazide diuretics, β-blockers, and calcium channel blockers can be used as adjuncts, though β-blockers should preferably be third-generation agents (nebivolol, carvedilol) with more favorable metabolic profiles (311, 323, 324).

## 8 Conclusion

**Figure 3:**
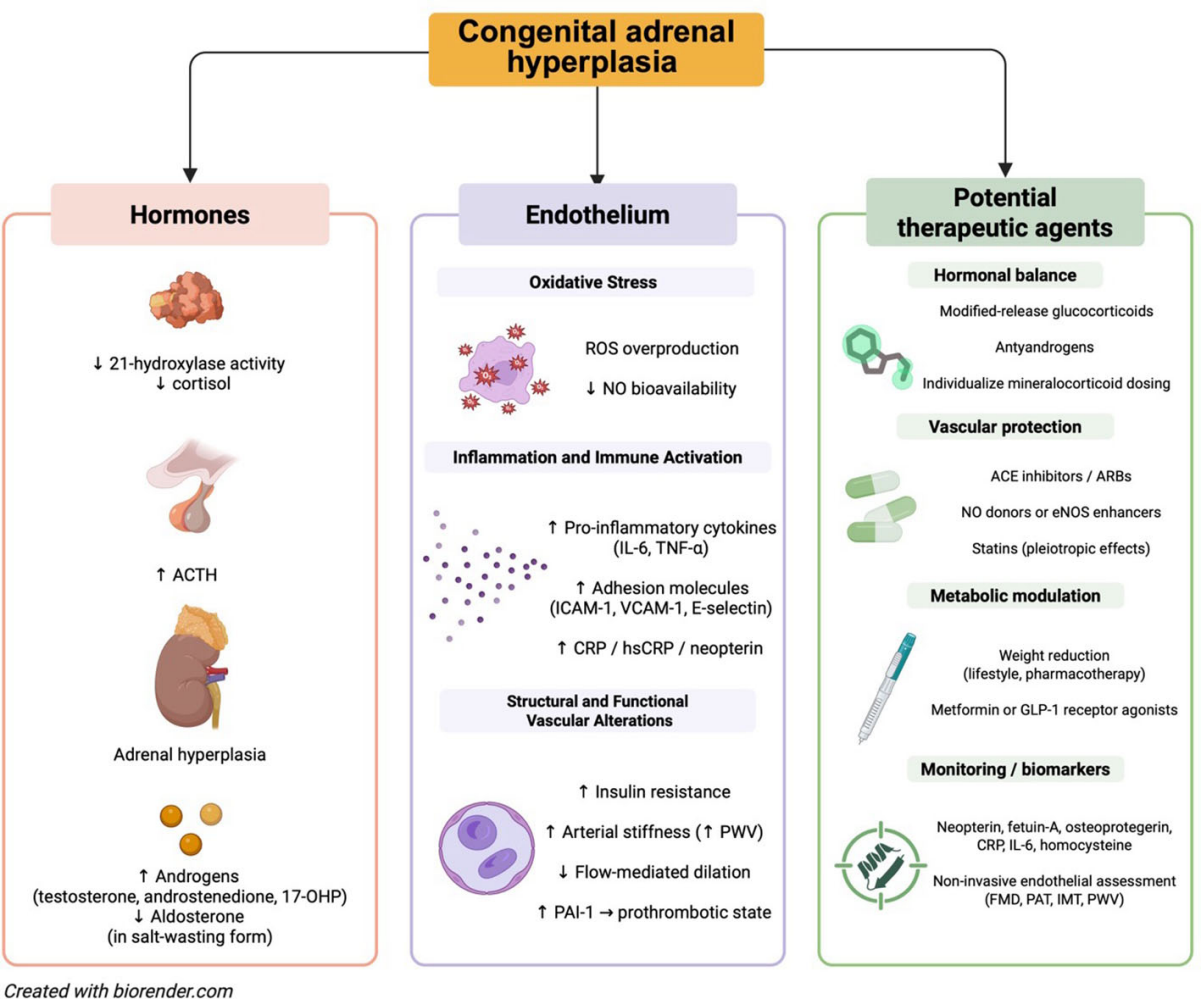
Pathophysiological links between hormonal dysregulation in CAH, endothelial dysfunction, and potential therapeutic targets. CAH, due to 21-hydroxylase deficiency, leads to impaired cortisol synthesis and compensatory ACTH overproduction, resulting in adrenal hyperplasia, androgen excess, and—particularly in salt-wasting forms—aldosterone deficiency. These hormonal disturbances may contribute to endothelial dysfunction through mechanisms such as increased oxidative stress, chronic inflammation, arterial stiffness, and impaired flow-mediated dilation. Therapeutic strategies focus on restoring hormonal balance, providing vascular protection, addressing metabolic dysregulation, and utilizing novel biomarkers and non-invasive vascular assessments to monitor cardiovascular risk. 17-OHP, 17-hydroxyprogesterone; ACE inhibitors, angiotensin-converting enzyme inhibitors; ACTH, adrenocorticotropic hormone; ARBs, angiotensin II receptor blockers; CRP, C-reactive protein; eNOS, endothelial nitric oxide synthase; FMD, flow-mediated dilation; GLP-1, glucagon-like peptide-1; hsCRP, high sensitivity C-reactive protein; ICAM-1, intercellular adhesion molecule 1; IL-6, interleukin-6; IMT, intima-media thickness; NO, nitric oxide; PAI-1, plasminogen activator inhibitor-1; PAT, peripheral arterial tonometry; PWV, pulse wave velocity; ROS, reactive oxygen species; TNF-α, tumor necrosis factor alpha; VCAM-1, vascular cell adhesion molecule 1.

Endothelial dysfunction plays a critical role in the cardiovascular comorbidities observed in individuals with CAH due to 21OHD. The complex interplay of disease-related factors, such as androgen excess, metabolic disturbances, and glucocorticoid therapy, contribute to the heightened cardiovascular and metabolic risk in this population. The pathophysiological mechanisms driving endothelial dysfunction in CAH are multifaceted, involving oxidative stress, inflammation, and altered vascular tone. Although various diagnostic techniques—such as FMD, PAT, PWV, and IMT measurements—offer valuable insights into endothelial function, the optimal *in vivo* assessment method remains an area of ongoing investigation (**Figure 3**).

Emerging biomarkers of endothelial dysfunction, including neopterin, osteoprotegerin, fetuin-A, homocysteine, leptin, adiponectin, PAI-1, CRP, IL-6, and CECs, provide additional understanding of the pathophysiology of endothelial damage. Elevated levels of these biomarkers have been documented in populations with cardiovascular risk factors, indicating their potential for early detection and monitoring of endothelial dysfunction in CAH. Despite their promise, several limitations impede their translation into routine clinical practice. The lack of standardized chemical assay protocols for evaluating endothelial damage, high cost, and technical complexity restrict their current application to research settings primarily. Furthermore, many endothelial biomarkers exhibit limited sensitivity and specificity, often overlapping with markers of inflammation, platelet activation, and vascular smooth muscle dysfunction. Small sample sizes in clinical studies further constrain the reliability of these biomarkers, necessitating large-scale, multicenter trials to validate their clinical utility. Some findings remain controversial or inconclusive, highlighting the need for standardized approaches to address discrepancies in the literature. Additionally, the impact of metabolic and cardiovascular comorbidities on endothelial biomarkers remains insufficiently explored, particularly in the context of multiple coexisting conditions, which may have additive or interactive effects on biomarker levels.

Until stronger evidence becomes available, strategies for cardiovascular risk reduction in CAH should follow existing recommendations for high-risk populations, focusing on aggressive management of hypertension, dyslipidemia, disturbances in glucose metabolism, and obesity. Pharmacological strategies to improve cardiovascular health in CAH include optimized glucocorticoid replacement, adjunctive antiandrogen therapies, insulin sensitizers, statins, and renin–angiotensin system inhibitors, although disease-specific evidence remains limited.

Nevertheless, the present analysis is constrained by several important methodological limitations that warrant careful consideration. Most of the studies included in this review are observational, often retrospective in nature, and are limited by small sample sizes. Additionally, various sources of bias, such as sampling bias, recall bias, observation bias, and confounding bias, further weaken the strength of the available evidence and complicate the interpretation of findings related to endothelial dysfunction in CAH. Future research is needed to further investigate novel biomarkers of endothelial dysfunction and to refine diagnostic strategies, potentially integrating endothelial function assessment, to enhance cardiovascular risk evaluation and management. Particularly, well-designed, prospective studies conducted on large cohorts and across different age groups are necessary to better understand biomarker dynamics over time and their predictive value in clinical practice. Longitudinal cohort studies are needed to track changes in endothelial function over time and to evaluate the effects of factors such as age, treatment duration, and adherence to medical regimens. Moreover, the application of omics-based technologies holds promise for the discovery of novel endothelial biomarkers in CAH, potentially enhancing risk stratification and personalized management. Such efforts are crucial to improving long-term cardiovascular outcomes in individuals with CAH. Special attention to vascular health is essential for children with CAH, as atherosclerotic processes typically initiate in childhood and progress more rapidly in high-risk populations (80).
